# Psychotrauma research in the Netherlands

**DOI:** 10.3402/ejpt.v4i0.20873

**Published:** 2013-05-02

**Authors:** Miranda Olff, Eric Vermetten

**Affiliations:** 1 Department of Psychiatry Academic Medical Center, University of Amsterdam & Arq Psychotrauma Expert Group, Diemen, The Netherlands; 2 Research Center—Military Mental Health, Ministry of Defense Utrecht, The Netherlands; 3 Department of Psychiatry University Medical Center Utrecht, Utrecht, The Netherlands

**Fig. 1:**
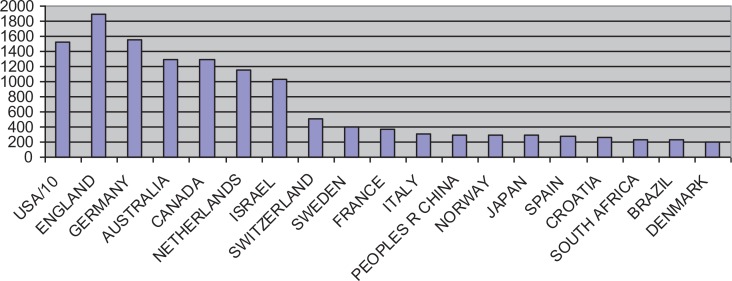
Total number of publications on traumatic stress*. *From Web of Science Citation Index Expanded (SCI-EXPANDED), Social Sciences Citation Index (SSCI), Arts & Humanities Citation Index (A&HCI), 1975–present, search performed March 2013. Topic=(“posttraumatic stress disorder”) OR Topic=(“posttraumatic stress”) OR Topic=(PTSD) OR Topic=(psychotrauma) OR Topic=(psychotraumatology).

In the Netherlands, there seems to be a relatively large interest in traumatic stress and its consequences. A literature search for publications in the field of psychotrauma and posttraumatic stress disorder (PTSD) in Web of Science shows that authors from the Netherlands are among the top seven in the world (see Fig. 1). Most research comes from the USA, and within Europe with the UK and Germany also being very active. Broadening the search terms does not change the patterns substantially. Correcting for the number of inhabitants as a proxy for the size of academic output, the Netherlands end up on second place, after Israel and shortly followed by Switzerland, Croatia, Norway and Australia. These are all countries with rather different backgrounds that ask for further analyses of the underlying reasons for their interest in the field.

In this thematic cluster we highlight psychotrauma research in the Netherlands. We start with a review of Dutch psychotraumatology (Vermetten & Olff, 2013), trying to find an answer to why there is this relatively high interest in trauma and its consequences in the country, describing major traumatic events and placing it in a historical context, for example, World War II. The other articles come from young researchers who have responded to a call to participate in a Master Class on psychotrauma research in the Netherlands. The candidates were nominated by their supervisors on the basis of content related to the field of psychotrauma research. The Master Class was held in Amsterdam at the Royal Netherlands Academy of Sciences on September 27, 2012. The three “masters” researchers who had been invited for the occasion were Professors Marc Creamer, Paula Schnurr and Rita Rosner. In a lively meeting, they commented on articles that were presented by this selected group of young professionals interested in psychotrauma. The results of this Master Class were inspiring and resulted in research articles that had profited from the stimulating discussions with the masters. Those articles that passed the regular journal peer review are now grouped together in this thematic cluster. It not only demonstrates to us that this formula is fruitful but also illustrates a variety of interesting studies undertaken in the Netherlands. We thank the masters for their initial comments when the articles were first presented. The authors have used these comments to strengthen their studies or analyses or change the designs. Also, it may be noted that there are many more high-quality research efforts by many other Dutch students and researchers—so by no means is this collection of articles a representative sample of Dutch research.

The aforementioned articles include an article by Diehle et al. (2013), who presented a study of a cross-cultural validation of the Clinician-Administered PTSD Scale for Children and Adolescents in a Dutch population. They looked at 112 children between the age of 8 and 18 that were recruited at 2 trauma-centers. Sleijpen et al. (2013) (Utrecht University and Arq) presented a review of resilience in young refugees and described a “mixed methods” approach to assess resilience in this population. Lommen et al. (2013) performed an experimental study and looked at the misinformation effect outside of the laboratory and factors contributing to it. A total of 249 soldiers were interviewed about stressors on deployment, but they also received misinformation about a fictional event on deployment. Seven months later, more than a quarter of the soldiers reported that they had actually experienced the fictional event. Meyer et al. (2013) performed a study in which they demonstrated that acute stress differentially affects spatial configuration learning in high and low cortisol responding healthy adults. Acute stress was induced in 34 participants by the Maastricht Acute Stress Test (MAST). The effects of stress and cortisol activity on subsequent spatial contextual cueing task (SCCT) performance were compared to SCCT performance following a no-stress control condition. Nijdam et al. (2013) studied neurocognitive functioning in patients with PTSD, with and without major depression, in 140 treatment-seeking outpatients who had a diagnosis of PTSD after various single traumatic events. The results of this study suggest that a more impaired neurocognitive profile may be associated with the presence of comorbid major depression, and verbal memory functions are discussed with reference to trauma-focused psychotherapy.

*Miranda Olff* Department of Psychiatry Academic Medical Center University of Amsterdam & Arq Psychotrauma Expert Group Diemen, The Netherlands*Eric Vermetten* Research Center—Military Mental Health Ministry of Defense Utrecht, The Netherlands Department of Psychiatry University Medical Center Utrecht Utrecht, The Netherlands
